# The hierarchy of stability and predictability in orthognathic surgery with rigid fixation: an update and extension

**DOI:** 10.1186/1746-160X-3-21

**Published:** 2007-04-30

**Authors:** William R Proffit, Timothy A Turvey, Ceib Phillips

**Affiliations:** 1 Departments of Orthodontics and Oral and Maxillofacial Surgery, School of Dentistry, University of North Carolina, Chapel Hill, NC 27599-7450, USA

## Abstract

A hierarchy of stability exists among the types of surgical movements that are possible with orthognathic surgery. This report updates the hierarchy, focusing on comparison of the stability of procedures when rigid fixation is used. Two procedures not previously placed in the hierarchy now are included: correction of asymmetry is stable with rigid fixation and repositioning of the chin also is very stable. During the first post-surgical year, surgical movements in patients treated for Class II/long face problems tend to be more stable than those treated for Class III problems. Clinically relevant changes (more than 2 mm) occur in a surprisingly large percentage of orthognathic surgery patients from one to five years post-treatment, after surgical healing is complete. During the first post-surgical year, patients treated for Class II/long face problems are more stable than those treated for Class III problems; from one to five years post-treatment, some patients in both groups experience skeletal change, but the Class III patients then are more stable than the Class II/long face patients. Fewer patients exhibit long-term changes in the dental occlusion than skeletal changes, because the dentition usually adapts to the skeletal change.

## Background

The Dentofacial Program at the University of North Carolina was begun in 1975 as a way to coordinate the evaluation and treatment of patients who needed orthodontics and orthognathic surgery, and as a way to facilitate research in this area. A research grant focused on the outcomes of orthognathic surgery at UNC, funded by the National Institute of Dental and Craniofacial Research, enters its 28^th ^year in June 2007.

This research project has resulted in more than 100 research papers in peer-reviewed journals, and about half that many invited contributions and book chapters. It became obvious by the 1990s that a major influence on the outcomes of orthognathic surgery was the amount and direction of surgical movement. A series of research papers that focused specifically on stability as related to the different surgical movements was summarized in 1996 in a paper outlining a hierarchy of stability related to surgical movements [1]. The purpose of this paper is to update the hierarchy by extending it to include treatment of asymmetries and provide further information with regard to long-term stability.

## Methods

The data base created through this project currently (February 2007) has records on 2264 patients who have had orthognathic surgery. Nearly twice that many have had initial records through the Dentofacial Program after they were referred for evaluation. Many of these were judged not to need surgery; the remainder did not accept it if it was recommended [2,3]. As of February 2007, at least one year follow-up is available for 1475 patients who did receive surgery, and five year or longer postsurgical follow-up is available for 507 patients.

Stability has been evaluated primarily from lateral cephalometric radiographs, which for all our studies have been oriented with the SN line rotated down 6° anteriorly, a position that approximates natural head position and is more reproducible than the Frankfort plane. This horizontal line is used as the x axis, and a vertical plane perpendicular to it through sella as the y axis, so that changes in landmark locations can be registered as x, y coordinate changes.

When stability is considered, it is important to keep in mind that there is not a normal distribution of post-surgical or post-treatment change. Instead, most of the changes occur in a few of the patients. Mean changes and standard deviations, therefore, can be misleading. The error in locating most cephalometric landmarks is less than 1 mm, and does not exceed 2 mm for any landmark. The hierarchy of procedures presented in this paper is primarily based on the number (percentage) of patients who experienced changes of at least 2 mm. We consider changes of <2 mm within the range of method error and clinically insignificant; 2–4 mm outside the range of method error and potentially clinically significant; and >4 mm as often beyond the range of orthodontic compensation and clinically highly significant.

The results presented below represent a compilation of stability data from the UNC database that have been reported previously in separate publications.

## Results

For the purposes of this extension of the hierarchy, it is important to differentiate post-surgical stability (changes in the first post-surgical year, which relate directly to the surgical healing, post-treatment orthodontics and short-term physiologic adaptation) from post-treatment stability (changes beyond one year post-surgery, which relate to long-term adaptation and for some patients, to post-treatment growth).

### The First Post-Surgical Year

**Figure 1:**
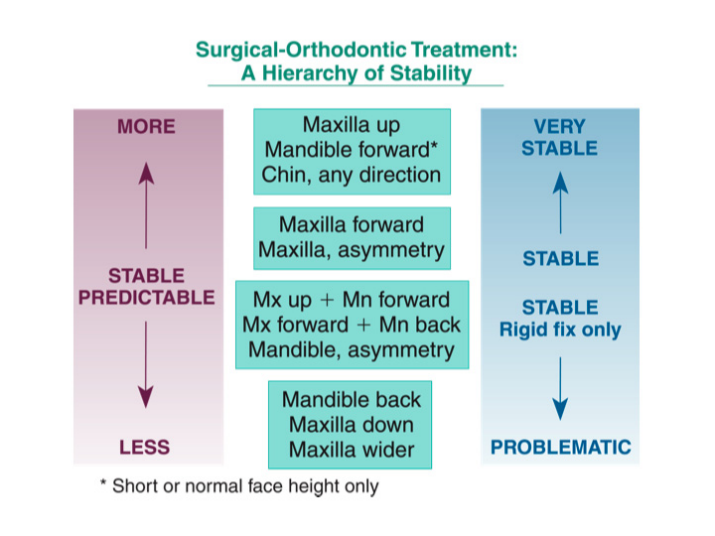
The extended hierarchy of stability, showing relative stability during the first postsurgical year.

A revised hierarchy for post-surgical stability (the first post-surgical year) is shown in Figure 1. Asymmetry and genioplasty have been added, and the surgical movements are grouped to emphasize the similarity of stability (percent of patients with >=2 mm changes) with different surgical procedures. The grouping simply reflects that differences between some procedures in the hierarchy are quite small, while other differences can be quite large. Considering the procedures as they are grouped:

#### Highly stable

It is interesting that the two single-jaw procedures used to correct skeletal Class II problems, superior repositioning of the maxilla and advancement of the mandible, fall into the highly stable category [4-11]. This was also true with wire fixation. It must be kept in mind, however, that mandibular advancement at UNC has been restricted to patients with short or normal face height. Early experience showed a lack of stability with ramus surgery to rotate the mandible at the osteotomy site so that the chin was moved up to close an anterior open bite, and we have used superior repositioning of the maxilla (with or without mandibular surgery) for these long face patients, so that the rotation occurred at the condyle instead.

**Figure 2:**
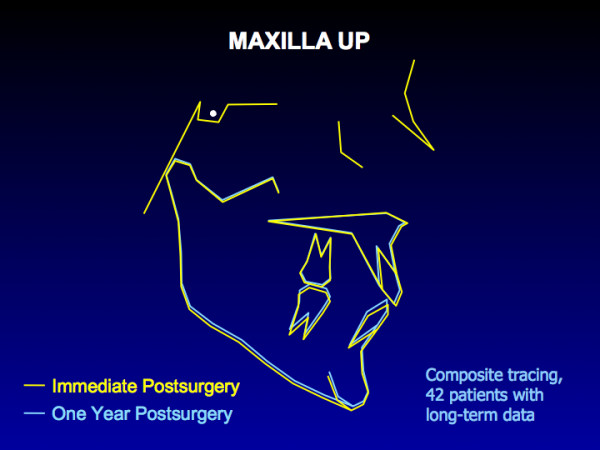
A composite tracing for 42 patients in whom the maxilla was moved up >2 mm. With this surgical movement and rigid fixation, there is almost no relapse tendency. The tracing shows a small upward movement from immediate postsurgery to one year that is due to removal of the surgical splint.

**Figure 3:**
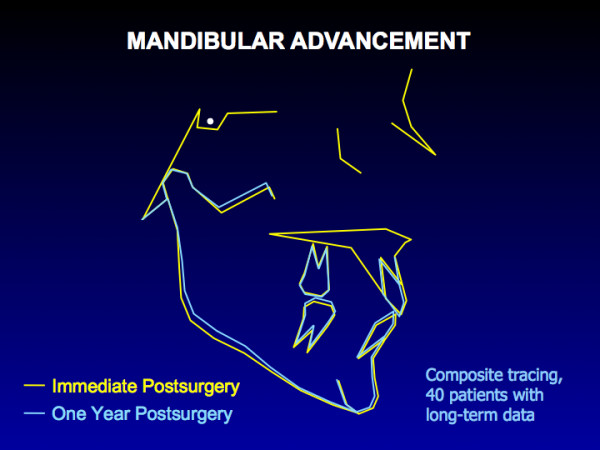
A composite tracing for 40 patients in whom the mandible was advanced >2 mm. The only significant change is a shortening of ramus height due to remodeling at the gonial angle, which is expected after a ramus osteotomy.

With rigid fixation, the maxilla is quite stable during the first postsurgical year when moved up (Figure 2), and there is almost no chance of clinically significant change. The composite tracing for the mandible (Figure 3) from immediate postsurgery to one year also shows almost no mean change in the horizontal position of the mandible, but the majority of the patients experience >2 mm upward movement of gonion due to remodeling in that area. Clinically, > 90% of the patients treated with either of these surgical procedures are judged to have excellent results.

Lower border osteotomy to reposition the chin also falls into the highly stable category, [12] with better remodeling of the symphysis noted in younger patients [13].

#### Stable

**Figure 4:**
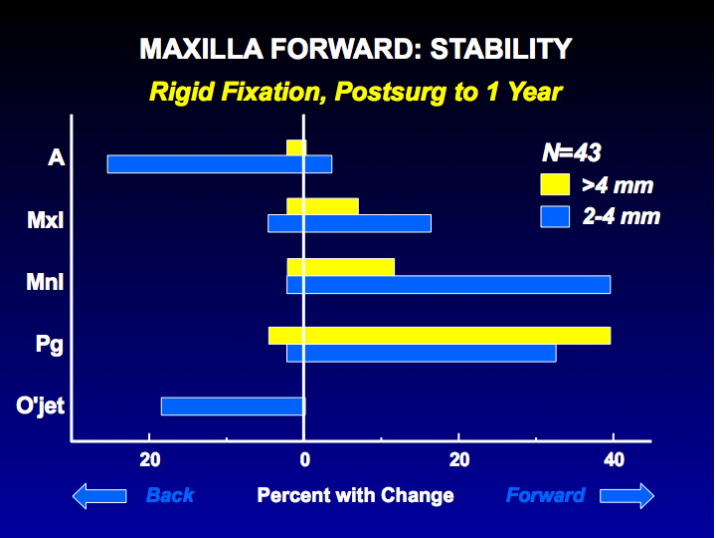
The percentage of patients with horizontal change in maxillary cephalometric landmark positions after forward movement of the maxilla and rigid fixation. Note that 20% of this group show mild relapse (2–4 mm backward movement of anterior maxillary landmarks), with almost no chance of clinically problematic relapse (>4 mm). Forward movement of mandibular landmarks reflects splint removal and a tendency for the maxilla to move upward if it was moved down as well as advanced.

Only one procedure, maxillary advancement, falls into this category [14,15]. The percentages for horizontal change with rigid fixation are shown in Figure 4. With or without rigid fixation, this translates into little or no change in the position of maxillary landmarks in about 80% of the patients, moderate relapse (2–4 mm change) in 20%, and greater relapse (>4 mm change) in almost none. As Figure 4 shows, post-surgical changes in the horizontal position of pogonion occur frequently in patients with maxillary advancement, because the mandibular rotates upward and forward when the surgical splint is removed.

#### Stable only with rigid fixation

Three procedures fall into this category: combined maxillary and mandibular surgery for correction of either Class II (maxilla up + mandible forward) or Class III (maxilla forward + mandible back) problems, and correction of facial asymmetry [8,16,17].

**Figure 5:**
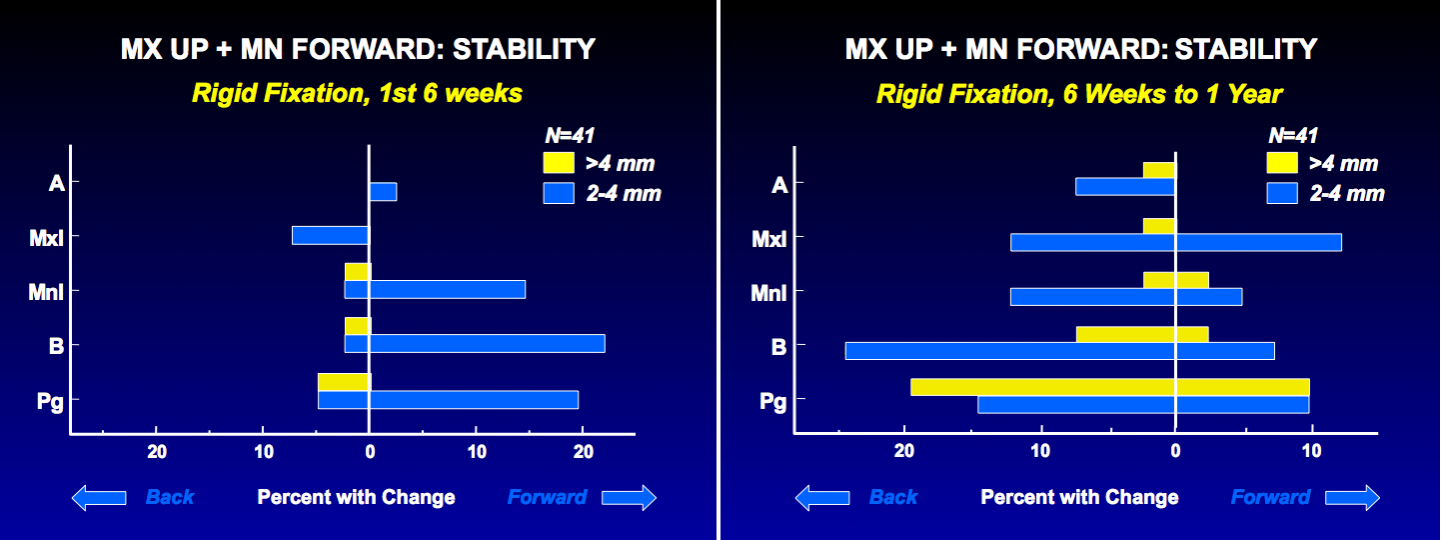
Stability after the combination of superior repositioning of the maxilla and advancement of the mandible: a, the percent of the patients with changes in the horizontal position of landmarks in the first 6 weeks postsurgery; b, the percent with changes from 6 weeks to 1 year.

For Class II patients, rigid fixation is needed for stability when both jaws are operated: the single jaw procedures are stable without rigid fixation but not when the procedures are combined. With rigid fixation, significant change (>2 mm) beyond what is created by mandibular rotation when the splint is removed occurs in only about 20% of the patients treated by a two jaw procedure (Figure 5). Clinically, an excellent result is obtained in 90% of the patients with rigid fixation, but in only 60% without it. A similar outcome is seen in 2-jaw Class III patients. Recent data show that stability with biodegradable plates and screws for rigid fixation is the same as with metal [18,19].

**Figure 6:**
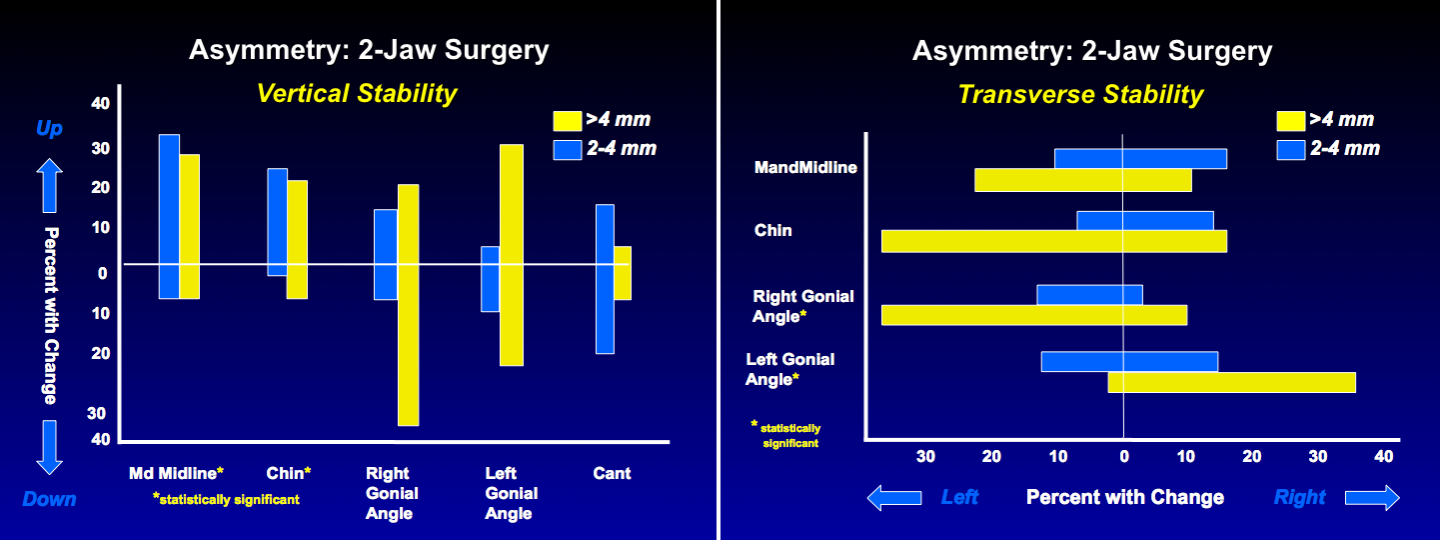
The percentage of patients with changes in landmark positions after two-jaw surgery to correct jaw asymmetry, using rigid fixation: a, vertical; b, transverse. Vertically asymmetric change in the position of the maxilla is quite stable. The dental midlines and chin show >2 mm transverse relapse in about one-third of the patients.

Correction of facial asymmetry usually also requires 2-jaw surgery, and rigid fixation facilitates obtaining a stable result. When the maxilla is repositioned vertically or horizontally in the correction of asymmetry, the relapse tendency is minimal (Figure 6a). Remodeling of the gonial angle is similar to the changes after any mandibular ramus osteotomy. Asymmetric advancement or setback of the mandible does carry with it a relapse tendency (Figure 6b). The chin tends to move back in the direction from which it was moved at surgery, and nearly 50% of the patients have >2 mm change.

#### Problematic

**Figure 7:**
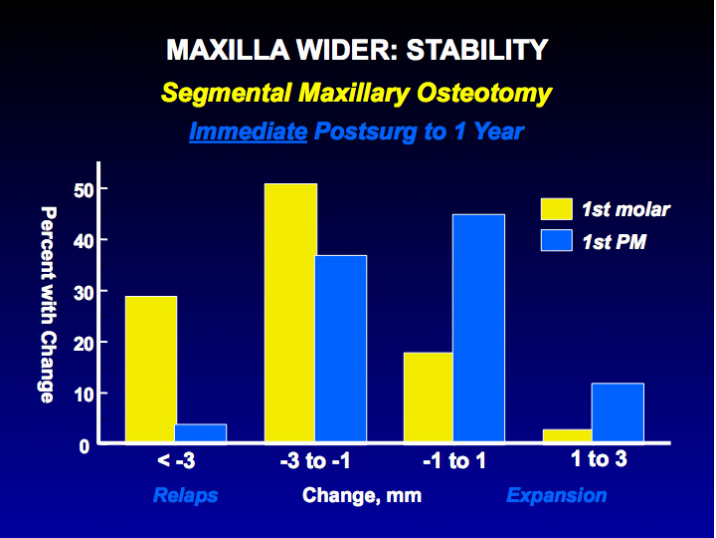
The percent of patients with changes following transverse expansion of the maxilla with segmental osteotomy. Greater expansion usually occurs at the molars than premolars with this procedure, and the percentage with relapse also is greater at the molars.

Three procedures fall into this category: isolated mandibular setback, [20] downward movement of the maxilla, [14] and widening of the maxilla [21]. For mandibular setback and downward movement of the maxilla without special fixation, up to 50% of the patients have >2 mm change, and up to 20% have >4 mm change. For widening of the maxilla (Figure 7), the amount of change is greater in the molar than premolar region but 30% have >3 mm relapse in expansion across the molars.

### Long-term Stability (Beyond One Year Post-Surgery)

A different pattern of stability exists when long-term post-treatment changes (changes between one and five years post-surgery) are considered [22-28]. After the first post-surgical year, when healing is complete, four interesting phenomena are observed: (1) in about 20% of the patients who had mandibular advancement (with or without simultaneous maxillary surgery), mandibular length decreases between 1 and 5 years post-treatment; (2) after superior repositioning of the maxilla, downward movement of the maxilla, in what appears to be a resumption of the original growth pattern, leads to >2 mm change in about one-third of the patients; (3) clinically significant changes in the position or dimensions of the maxilla and mandible occur in about twice as many patients as similar changes in overjet or overbite; and (4) the Class III patients who tended to be less stable than Class II patients in the first post-surgical year show less change thereafter. Considering these in turn:

#### Changes in mandibular length: long-term condylar remodeling

**Figure 8:**
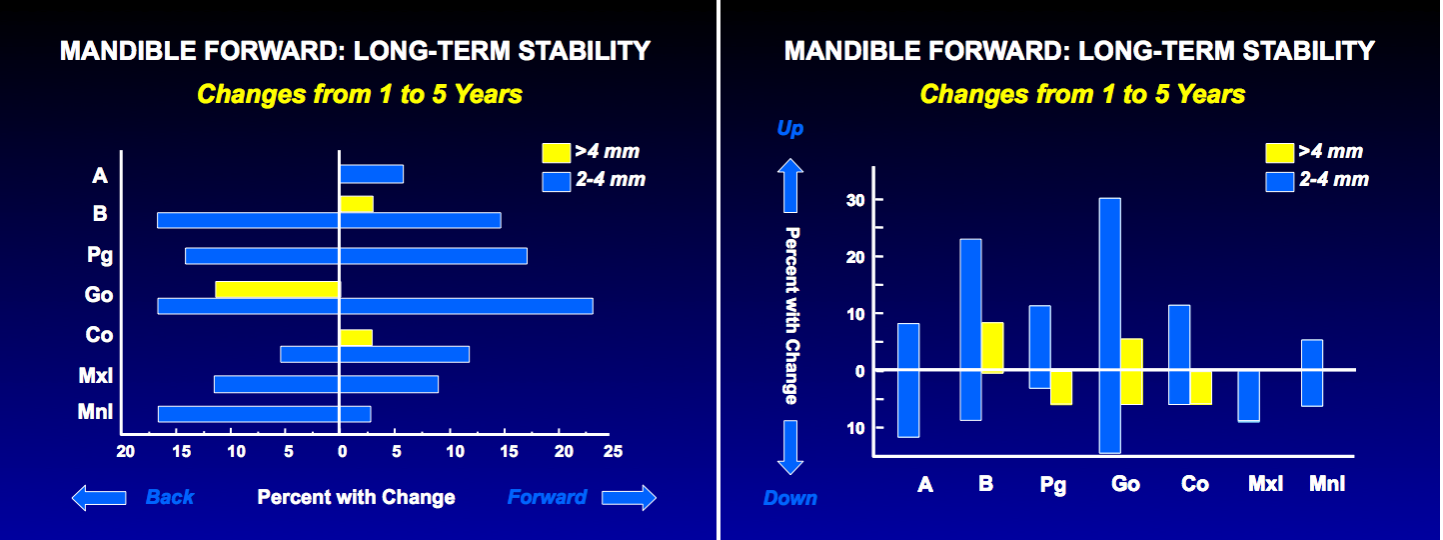
Changes from one year to 5 years after mandibular advancement: a, the percentage of patients with changes in the horizontal position of landmarks; b, the percentage with changes in vertical position. Points B and Pg are as likely to move forward as backward long-term. Beyond one year postsurgery, one-third of the patients continue to experience backward and upward movement of gonion, indicating a loss of bone at the gonial angle as remodeling continues, but 20% have a net gain.

Figure 8 shows long-term changes in the a-p position of the mandible after advancement. The data suggest that long after surgical healing is complete, remodeling at the mandibular condyles decreases mandibular length and ramus height in about 25% of the patients. An increase in overjet occurs in less than half the patients who experience this, because dental adaptation to the long-term change, primarily a proclination of the lower incisors, also occurs.

**Figure 9:**
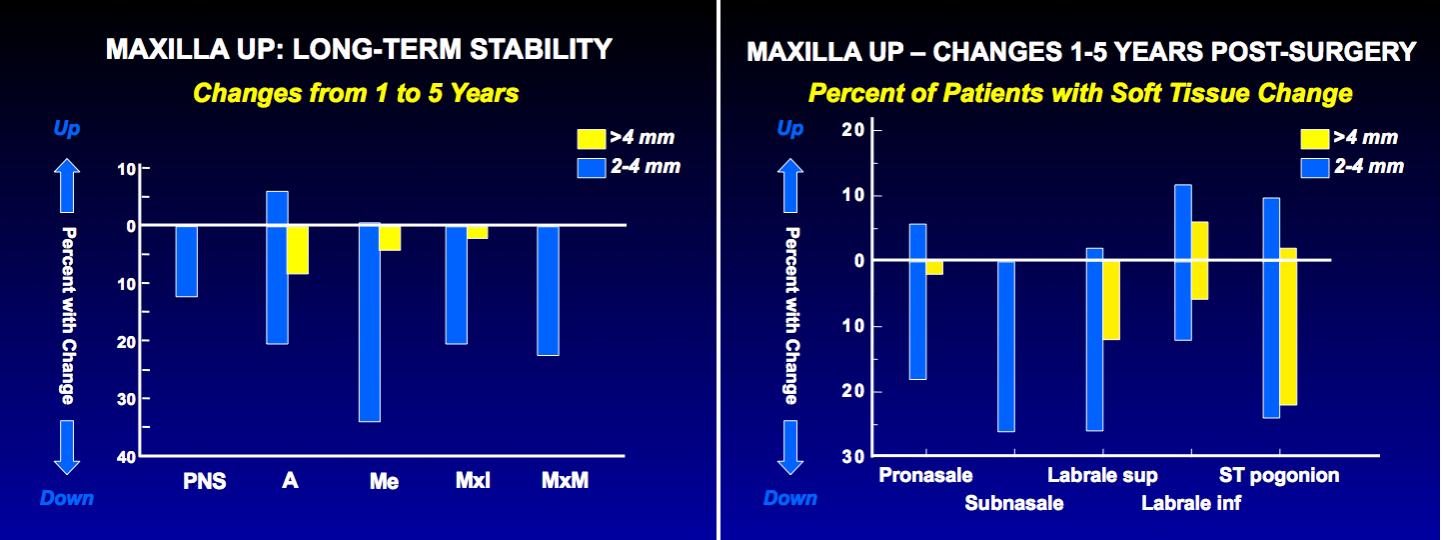
Changes from one year to 5 years after superior repositioning of the maxilla: a, the percentage of patients with changes in the vertical position of skeletal and dental landmarks; b, the percentage with changes in the vertical position of soft tissue landmarks. Although the long-term position of the maxilla is quite stable in 80% of the patients, 20% experience a downward movement, and when the downward movement occurs, parallel changes in the facial soft tissues occur.

**Figure 10:**
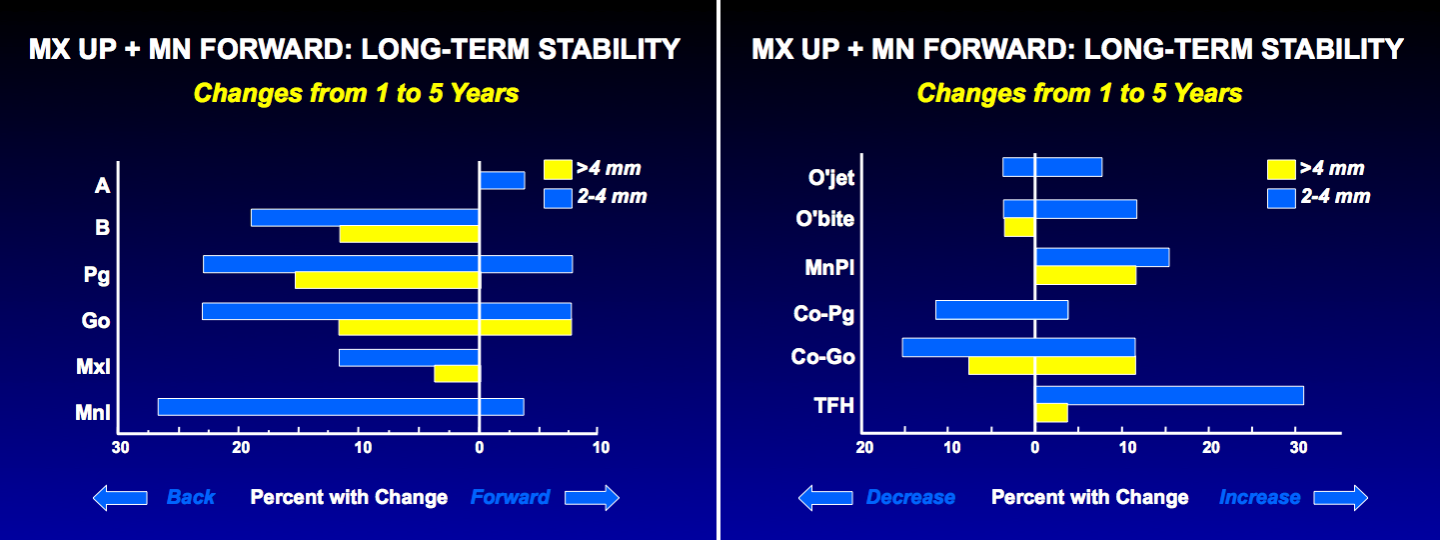
Changes from one to 5 years after two-jaw surgery for Class II problems: a, the percentage of patients with changes in the horizontal position of landmarks; b, the percentage of patients with changes in linear dimensions and the mandibular plane angle (TFH = total face height). Note that one-third of the patients experienced >2 mm backward movement of points B and Pg, and half of these had >4 mm decrease, and one-third had >2 mm downward movement of the maxilla, but overjet increased >2 mm in only 8% and >4 mm in none. This reflects a forward movement of the teeth relative to the mandible in compensation for the skeletal change. The Co-Pg distance decreased >2 mm in 12%, with no decrease >4 mm.

Figure 9 shows long-term changes in the vertical position of the maxilla, and long-term vertical and horizontal changes after 2-jaw surgery for Class II patients are shown in Figure 10. Note the large percentage of patients who had downward movement of the maxilla long after surgical healing was complete. It is interesting that soft tissue changes parallelled the downward movement of the bony structures (Figure 9b). In Figure 10, note also the similarity of the changes in 2-jaw surgery to those seen with isolated mandibular or maxillary surgery. Although it has been suggested that long face patients treated with 2-jaw surgery are particularly susceptible to long-term condylar remodeling, our data do not support this contention. The long-term changes in the position of the maxilla and the associated soft tissue changes seem to reflect a resumption of growth pattern at a time in life that it is not expected. As with the long-term mandibular changes that do not result in changes in overjet, the number of patients with clinically significant post-treatment bite opening is smaller than the number with late downward growth.

## Discussion

### Problematic Post-surgical Stability: Why?

**Figure 11:**
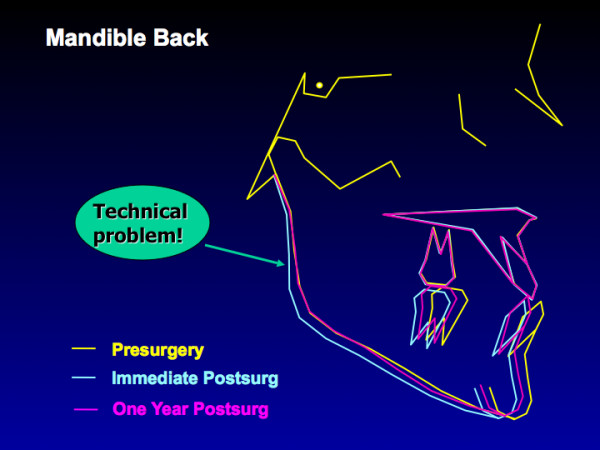
Composite superimpositions of a group of 19 patients with mandibular setback done before 1995. Note the backward movement of the ramus from pre- to post-surgery, and the return of the inclination of the ramus to its original position at one year – which carries the chin forward. Controlling the inclination of the ramus at surgery seems to largely eliminate relapse after mandibular setback.

With mandibular setback, problematic post-surgical stability likely is a technical problem. In a prognathic patient whose mandible is long, the objective of surgery is to move the chin closer to the gonial angle. At surgery, if the chin is moved back but the gonial angle also is pushed back, the musculature usually returns the ramus to its original orientation, and the chin is carried forward (Figure 11) [29]. The stability of two-jaw Class III treatment in the last decade provides some evidence that the technical problem in setting mandibles back has largely been overcome.

**Figure 12:**
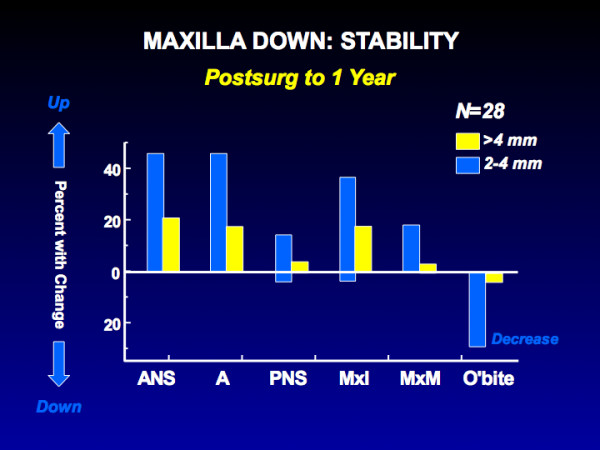
The percentage of patients with changes in the vertical position of the maxilla from immediate post-surgery to one year. Note that despite rigid fixation, nearly two-thirds of the patients had >2 mm upward movement of the anterior maxilla landmarks and 20% had >4 mm change. Moving the maxilla down is much more stable when a simultaneous ramus osteotomy is done (the preferred approach at UNC) or when a rigid interpositional graft is placed.

Problematic stability in moving the maxilla down is due largely to changes within the first few postsurgical weeks, before bone healing is complete, as occlusal force tends to push it upward (Figure 12). There are three logical approaches to maintaining the position of the maxilla until it heals: heavy rigid fixation, a rigid hydroxyl apatite graft in the defect created by the downward movement, and simultaneous mandibular surgery to decrease the occlusal force. All are reasonably successful, but the rigid fixation has to be much heavier than typical plates and screws and still is not completely effective. An initially rigid but ultimately resorbable graft, rather than one like hydroxyl apatite that persists indefinitely, is likely to become available in the near future and would be preferred. Improved stability has been demonstrated in patients (usually Class III) in whom downward movement of the maxilla is combined with a mandibular ramus osteotomy.

Widening the maxilla with a segmental osteotomy stretches the palatal soft tissues, and this tissue elasticity provides a force to decrease the expansion post-surgically (see Figure 6). Surgically-assisted expansion (SARPE), with a jackscrew in place across the palate to provide somewhat slower expansion and (perhaps more importantly) rigid retention, is a reasonable alternative if only transverse changes are needed. Are two surgical procedures, first SARPE and then a later one-piece LeFort I osteotmy, indicated instead of a one-stage segmental LeFort I when three-dimensional movements are needed [30][31]? The major reason for 2-stage surgery would be presumed better stability for expansion with SARPE, and a current study with better methodology than previous publications shows no significant differences between long term stability of expansion with osteotomy or SARPE [32]. Significant differences have not been documented between the outcomes of two-stage and one-stage approaches, but good data for this comparison do not yet exist.

### Long-term Post-treatment Stability

Beyond one year, changes are only indirectly related to surgery. Skeletal changes over a 5 year period can be shown in patients who did not have orthognathic surgery, [33] but in post-treatment orthognathic surgery patients, the changes tend to be larger [34]. In this time period, changes reflect adaptive bone remodeling and/or a resumption of growth, and adaptive changes in the dentition.

The data show that after Class II surgery, in patients who have long-term changes, there usually is a smaller increase in overjet than the decrease in mandibular length. Adaptation of the dentition to skeletal change, primarily proclination of the lower incisors, largely prevents the same degree of change in overjet. The same thing is seen in long face patients, many of whom had an anterior open bite, in whom long-term downward movement of the maxilla occurred. There was not the same degree of bite opening, because of compensatory eruption of the anterior teeth in both arches.

It is surprising that a smaller percentage of patients treated surgically for Class III problems have long-term changes than those treated for Class II problems. Because mandibular prognathic patients often have mandibular growth until an older age than individuals who do not have this problem, it would seem reasonable that continued mandibular growth long-term after surgery might occur, and that this would be more likely in those who had mandibular setback surgery at a younger age. The data do not support either of those ideas [35,36]. Beyond one year post-surgery, very few patients have forward growth of the mandible. Girls who had setback surgery before age 18, and boys who had it before age 20, were no more likely to have long-term mandibular growth than those treated at later ages.

## Conclusion

Data now exist to document the stability of changes in jaw position from orthognathic surgery. From the perspective of stability during the first post-surgical year, the surgical movements can be placed in four groups ranging from highly stable to problematic. The procedures typically used to treat Class II/long face problems are quite stable in the first year, the procedures typically used to treat Class III problems less so. A surprisingly large number of patients experience skeletal changes from one to five years post-surgery, when healing is complete, and in that time frame clinically relevant (>2 mm) changes are more likely in Class II/long face patients than in Class III patients. Fewer patients exhibit long-term changes in the dental occlusion than skeletal changes, because adaptive changes often occur in the dentition as skeletal changes occur. In both the post-surgical and post-treatment periods, almost all the changes occur in a minority of patients, so it is better to consider the percentage of patients with clinically significant changes than the mean changes. The database makes it clear that clinically satisfactory results can be obtained and maintained long-term in the great majority of orthognathic surgery patients, but the differences among various directions of movement must be taken into account when treatment is planned.

## Competing interests

The author(s) declare that they have no competing interests.

## Authors' contributions

Dr. Proffit served as principal investigator on the research grant that supported this work, and prepared the first draft of the manuscript. Dr. Phillips supervised the development of the project's data base, and was responsible for all statistical analyses. Dr. Turvey performed almost all the surgery for these patients and played a major role in gathering the clinical data on short- and long-term recalls. All three authors were involved in revision and final preparation of the manuscript.
